# Data associated with the characterization and presumptive identification of *Bacillus* and related species isolated from honey samples by using HiCrome *Bacillus* agar

**DOI:** 10.1016/j.dib.2019.104206

**Published:** 2019-07-02

**Authors:** Adriana M. Alippi

**Affiliations:** CIDEFI - Facultad de Ciencias Agrarias y Forestales, Universidad Nacional de La Plata, calles 60 y 119 S/N, 1900 La Plata, Buenos Aires, Argentina

**Keywords:** HiCrome bacillus agar, Honey, *Bacillus*, *Brevibacillus*, *Lysinibacillus*, *Paenibacillus*, *Rummeliibacillus*, Aerobic spore-forming bacteria, Chromogenic media

## Abstract

The dataset described in this paper provides information on the morphological features of 24 different species of the genera *Bacillus, Paenibacillus, Brevibacillus, Lysinibacillus,* and *Rummeliibacillus*when growing in HiCrome Bacillus agar. The species studied are common contaminants of honey. In support to the recent publication entitled “HiCrome Bacillus agar for presumptive identification of Bacillus and related species isolated from honey samples” (2), a collection of 197 bacterial isolates belonging to 24 different species of aerobic spore-forming bacteria have been screened for their colony appearance and color and any substrate color change of HiCrome Bacillus agar at 24 and 48 h of incubation. Two simple flowcharts utilizing a combination of colony and media characteristics in the chromogenic medium and a set of simple biochemical and morphological tests were developed for quick presumptive identification.

**Table:** Specifications table

Subject area	Microbiology, Food Microbiology
More specific subject area	Microbiological Methods, Bioinformatics
Type of data	Tables, Figures, Flowcharts
How data was acquired	Digital camera, PCR, purification, sequencing, and Phylogenetic analysis
Data format	Analyzed
Experimental factors	Isolation of spore-forming bacteria Genomic DNA from pure bacterial cultures
Experimental features	Isolation and cultivation of bacteria, 16S rRNA sequencing, microbiological tests, colony morphology, and microscopy
Data source location	Bacteria were isolated from samples from different geographical areas The analysis was performed at CIDEFI - Facultad de Ciencias Agrarias y Forestales, Universidad Nacional de La Plata, Calles 60 y 119 S/N, 1900 La Plata, Buenos Aires, Argentina
Data accessibility	Data are available with this article. 16S rRNA sequences of selected bacterial strains (n = 56) isolated from honey or honeybee larvae have been deposited in GenBank (https://www.ncbi.nlm.nih.gov/genbank/) under accession numbers summarized in Table 1.
Related research article	HiCrome Bacillus agar for presumptive identification of *Bacillus* and related species isolated from honey samples by Alippi and Abrahamovich (*International Journal of Food Microbiology, 2019, DOI: 10.1016/j.ijfoodmicro.2019.108245)*

**Table:** 

**Value of the data** • Data presented to describe the colony appearance on HiCrome Bacillus agar of several *Bacillus* and related species commonly found in honey. • Data generated serve as a point of reference for further research in microbial diversity, microbial ecology, and microbial taxonomy. • The information presented could be beneficial for other researchers that are interested in the microbiology of honey and is potentially also of benefit for research on other food products.

## 1 Data

The dataset described in this paper provides information on the morphological features of 24 different species of the genera *Bacillus, Paenibacillus, Brevibacillus, Lysinibacillus,* and *Rummeliibacillus* when growing in HiCrome Bacillus agar. The species studied here have been previously reported in honey [1], [2], [3], [5], [7], [10], [12], [13], [14], [15].

**Fig. 1:**
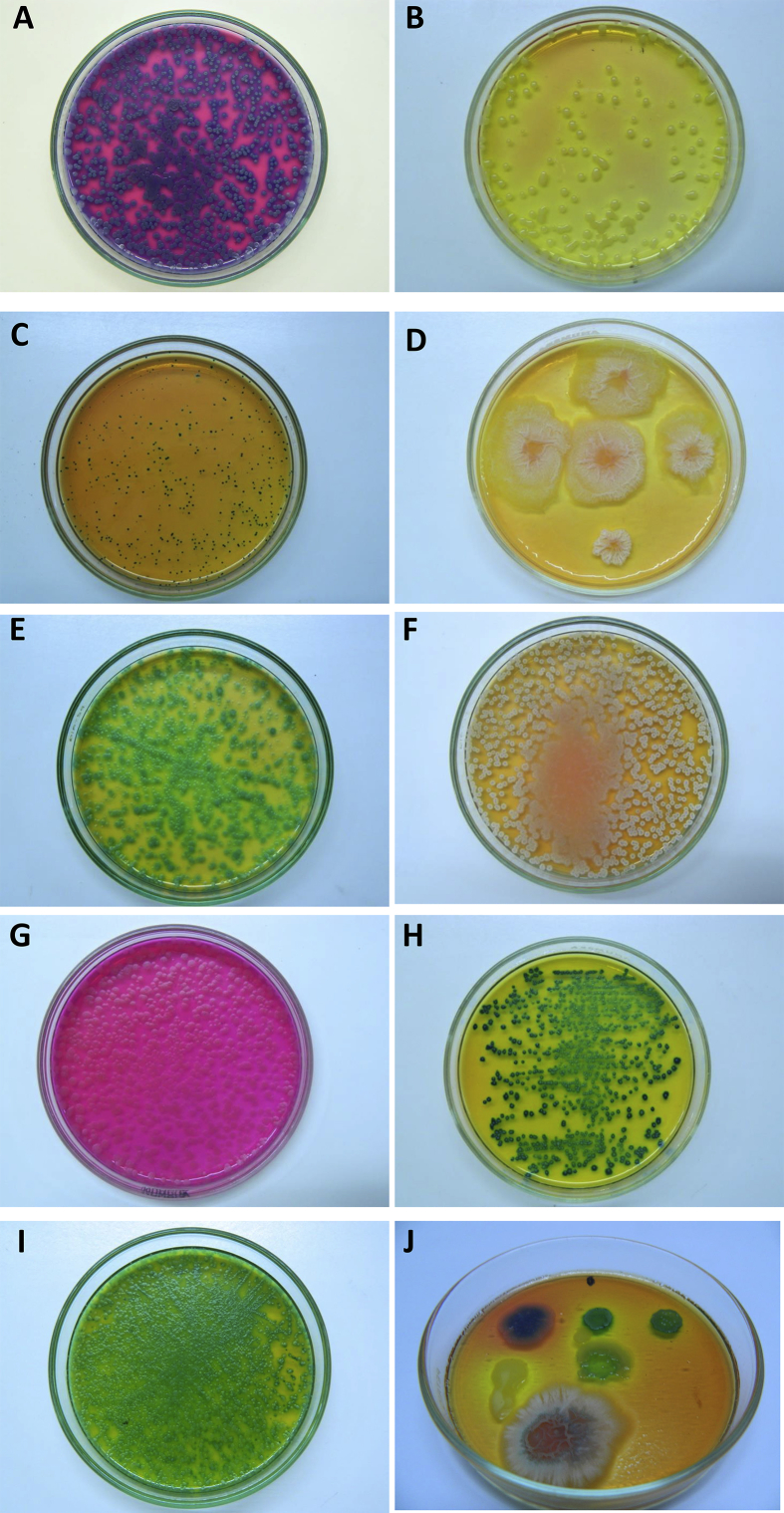
Comparison of colony appearance on HiCrome Bacillus agar of several *Bacillu*s and related species commonly found in honey: A. *Bacillus cereus* m87, B. *Bacillus megaterium* m344, C. *Paenibacillus alvei* mv82, D. *Bacillus amyloliquefaciens* m39, E. *Bacillus subtilis* ATCC 7061*,* F. *Bacillus subtilis* m191, *G. Lysinibacillus sphaericus* m533, *H. Bacillus licheniformis* mv68, *I. Bacillus pumilus* mv49b, J. Complex sample of naturally contaminated honey containing (Clockwise from upper left) *B. cereus, B. licheniformis, B. pumilus B. amyloliquefaciens,* and *B. megaterium.*

A collection of 197 bacterial isolates belonging to the 24 species tested have been screened for their colony appearance and color and any substrate color change of HiCrome Bacillus agar at 24 and 48 h of incubation (Fig. 1 and Table 3). Colors of colonies and substrate observed were compared with a Pantone international chart and identified with a PMS number (http://www.cal-print.com/InkColorChart.htm).

**Fig. 2:**
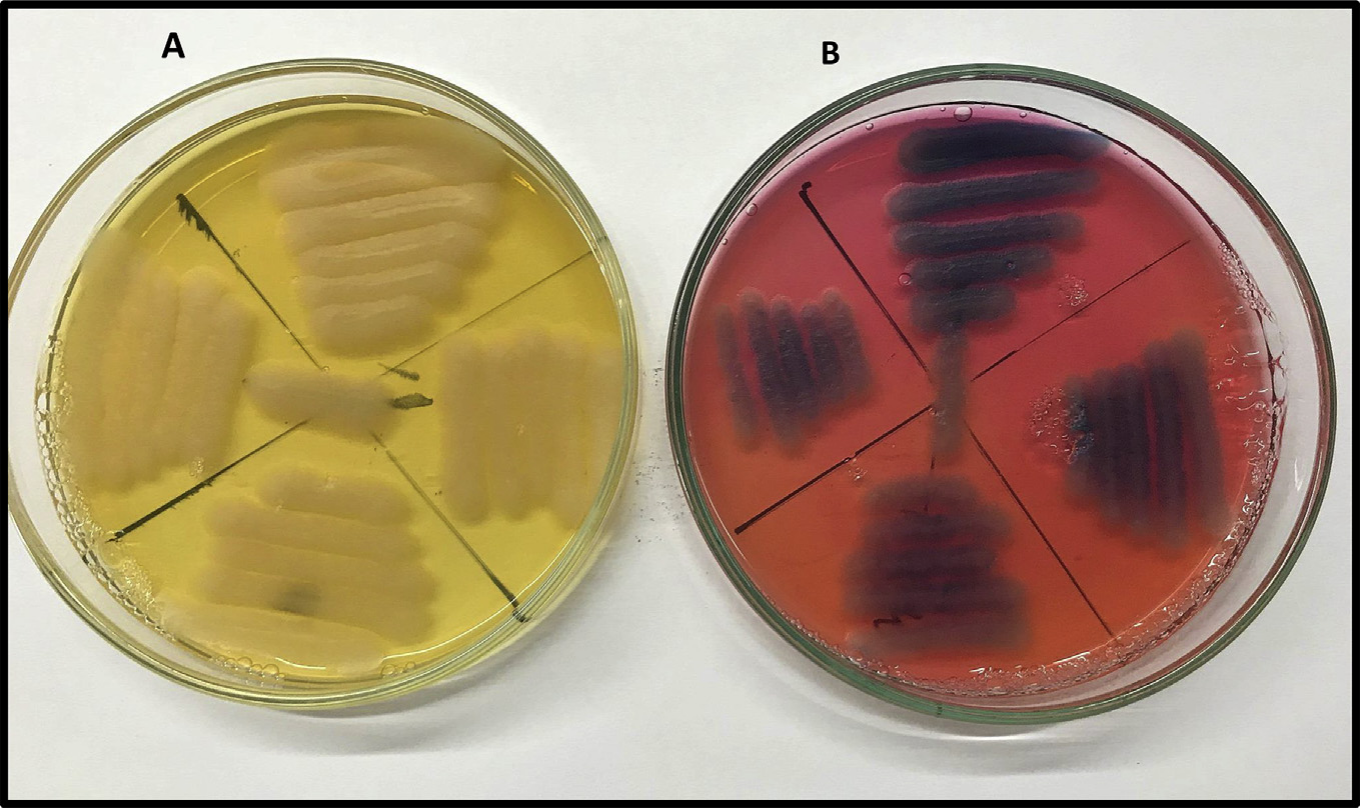
*Bacillus cereus* ATCC 11778 growing on A: BHIT and B. HiCrome Bacillus agar showing luxuriant growth (+++) and E value = 5.

**Fig. 3:**
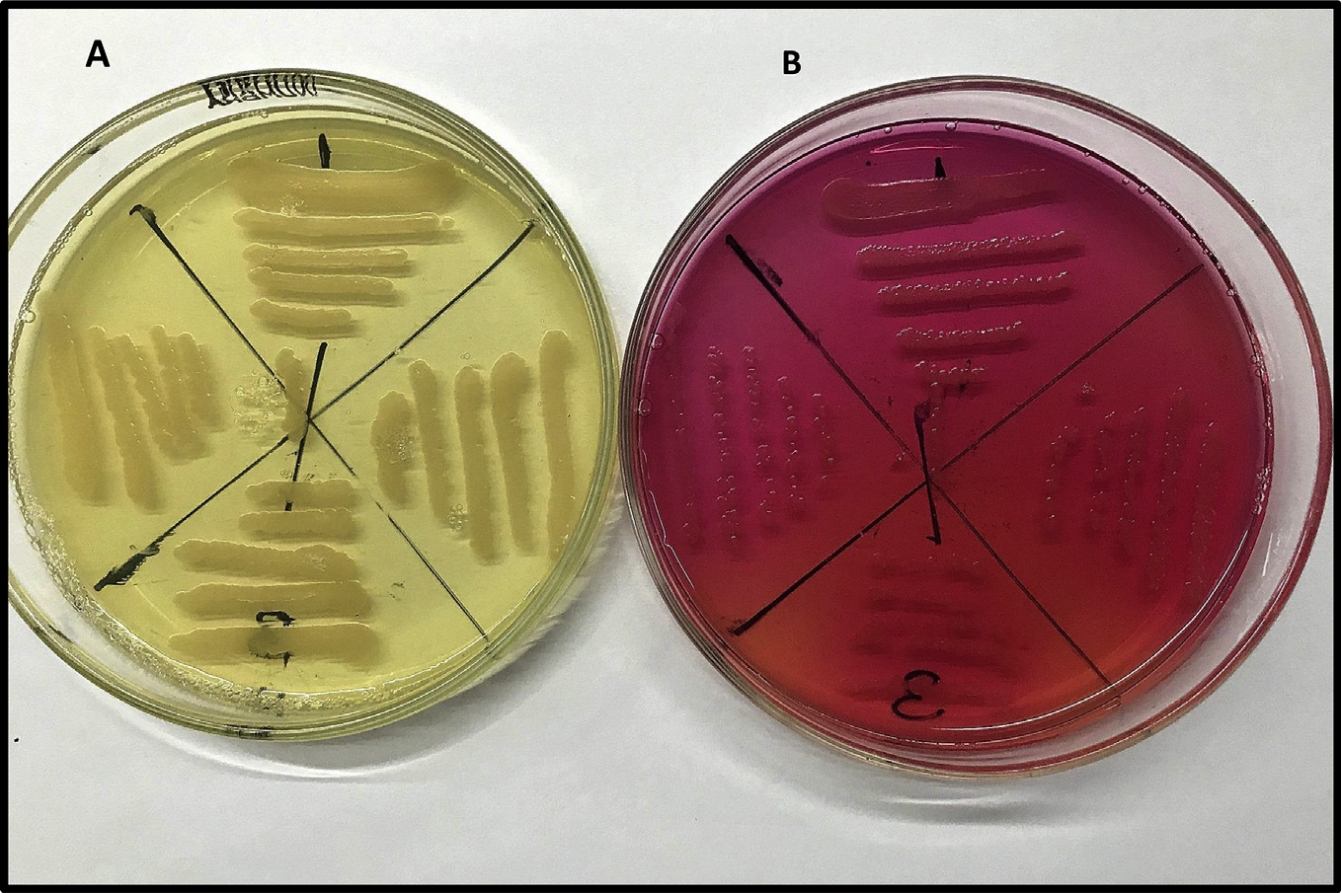
*Rummeliibacillus stabekisii* mv111 growing on A: MYPGP and B. HiCrome Bacillus agar showing good growth (++) and E value = 5.

The Ecometric technique was used for comparative evaluation of HiCrome Bacillus agar and the control medium (Fig. 2, Fig. 3 and Table 3). E-values (Table 3) were obtained for 28 selected isolates tested by using previously published methods [2], [8].

**Fig. 4:**
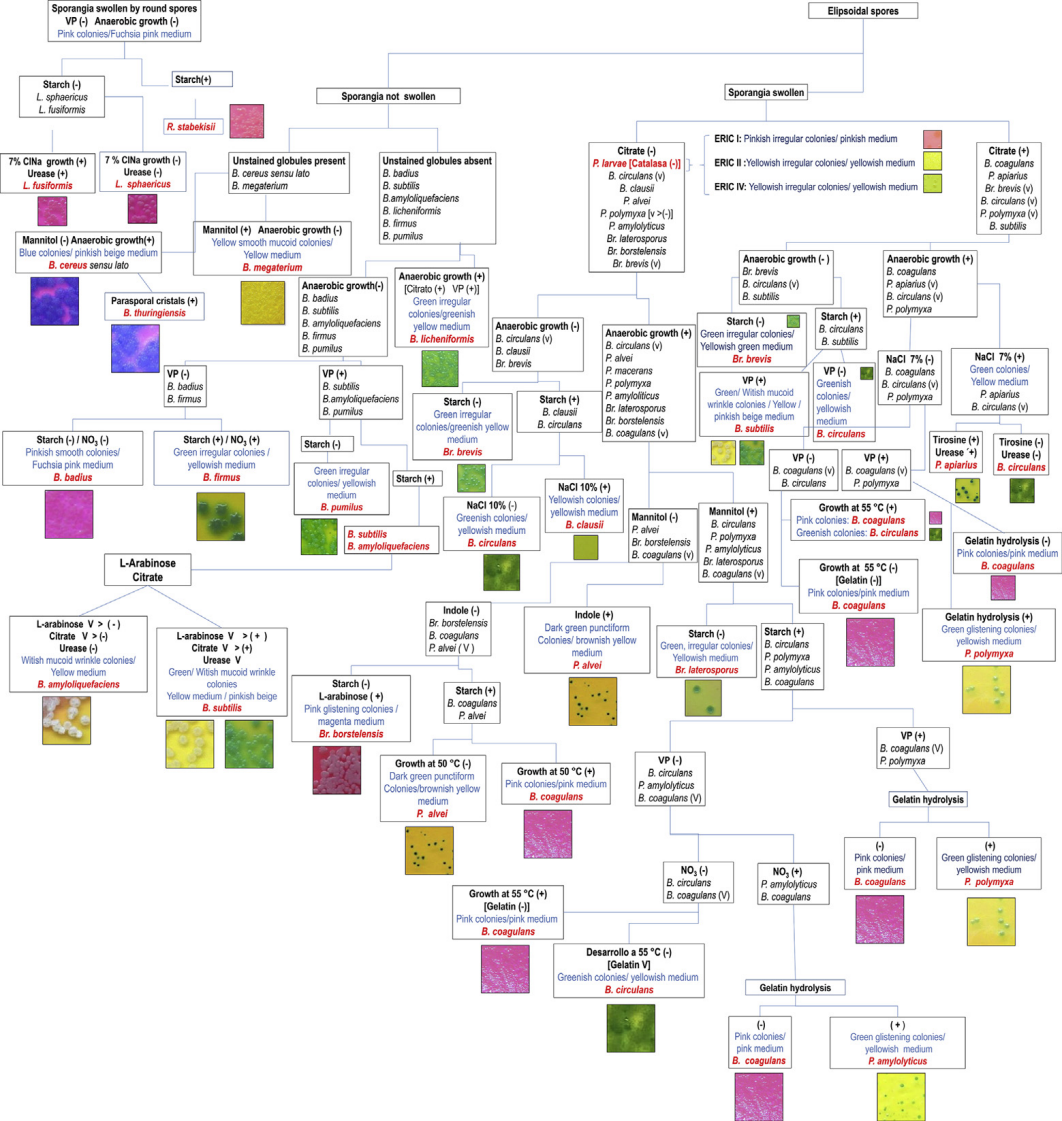
Flowchart - Main steps for the identification of common strains of *Bacillus* and related species from honey by using a combination of selected morphological and biochemical tests and HiCrome Bacillus agar.

**Fig. 5:**
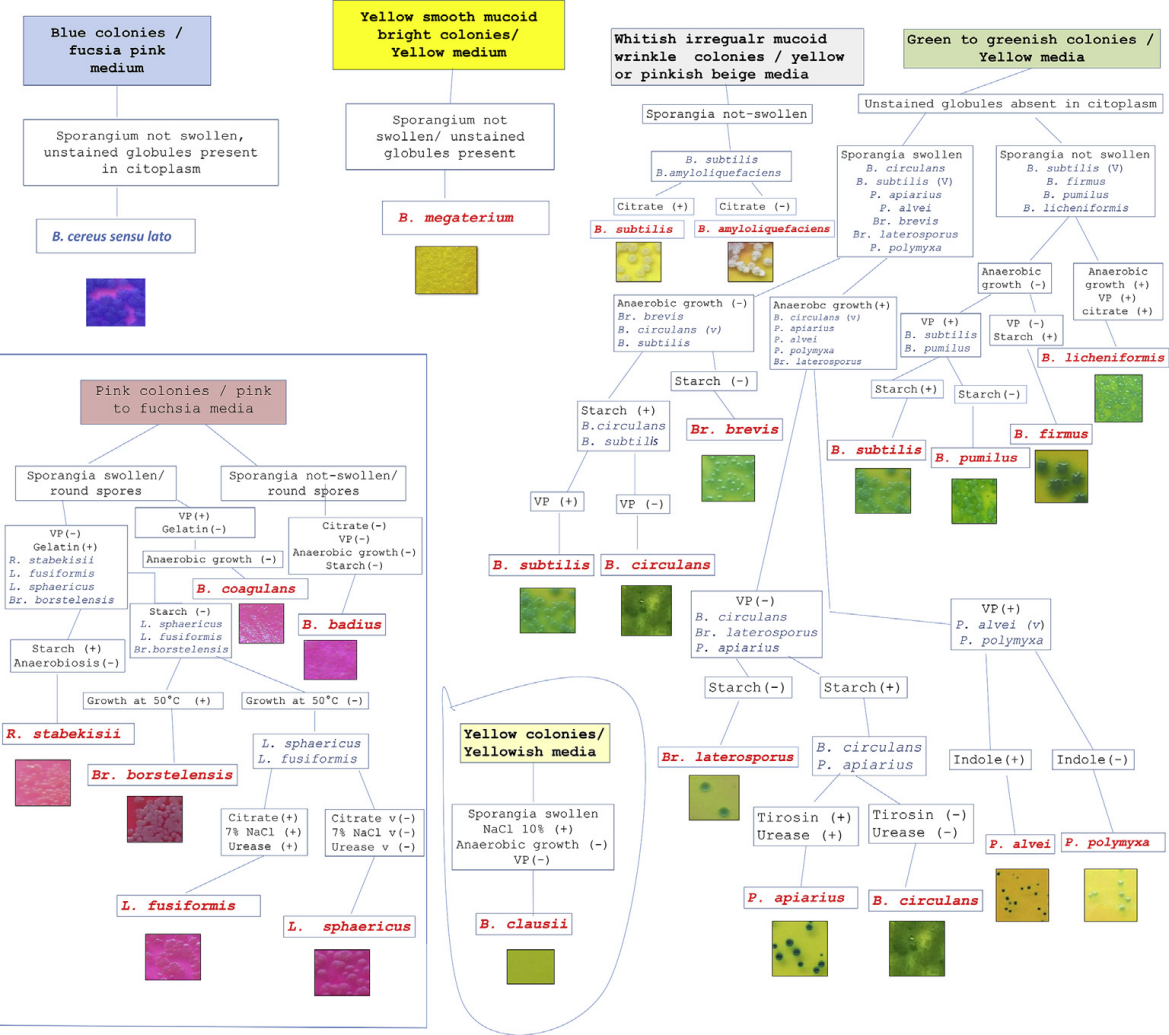
Flowchart - Simplified steps for the identification of common strains of *Bacillus* and related species from honey by using isolation in HiCrome Bacillus agar and selected morphological and biochemical tests.

Two Flowcharts were prepared by a combination of colony and media characteristics in HiCrome Bacillus agar and a set of selected biochemical and morphological tests that are used routinely in Microbiological laboratories. The first chart (Fig. 4) permits the identification of the aerobic spore-forming bacteria reported in honey by a few simple tests. The more simplified flowchart presented in Fig. 5 allows differentiating typical strains of aerobic spore-forming species by direct isolation from honey.

The bacterial identity of selected strains isolated from honey or honeybee larvae (n = 56) (Table 1) were confirmed by sequencing the 16S rDNA. Sequences were deposited in the DDBJ/EMBL/Genbank under the Accession Numbers listed in Table 1. For comparisons, 16S rRNA sequences from type cultures (n = 32 plus 1 outlier) were used and are listed in Table 2.

**Table 1:** Source and accession numbers of bacterial strains and isolates used in this study.

Species	Strain/Isolate designation	Source and geographical origin	Accesion number
*Bacillus amyloliquefaciens*	xx	Honeybee larvae-Argentina	KP177517.1
*Bacillus amyloliquefaciens*	mv35	Honey- Argentina	MG004186.1
*Bacillus amyloliquefaciens*	m39	Honey- Argentina	MG004187.1
*Bacillus amyloliquefaciens*	m163b	Honey- Argentina	MG004188.1
*Bacillus amyloliquefaciens*	m164b	Honey- Argentina	MG004193.1
*Bacillus amyloliquefaciens*	m287b	Honey- Argentina	MG004189.1
*Bacillus amyloliquefaciens*	m291b	Honey- Argentina	MG004190.1
*Bacillus badius*	CCT 0196	CCT	N/A
*Bacillus cereus*	ATCC 11778	ATCC	AF290546.1
*Bacillus cereus*	cm4	Honey- Argentina	N/A
*Bacillus cereus*	m6c	Honey- Argentina	KP005456.1
*Bacillus cereus*	cm7	Honey- Argentina	N/A
*Bacillus cereus*	cm8	Honey- Argentina	N/A
*Bacillus cereus*	m9a	Honey- Argentina	N/A
*Bacillus cereus*	m10a	Honey- Argentina	N/A
*Bacillus cereus*	m10b	Honey- Argentina	N/A
*Bacillus cereus*	m12	Honey- Argentina	N/A
*Bacillus cereus*	m19	Honey- Argentina	N/A
*Bacillus cereus*	m21	Honey- Argentina	N/A
*Bacillus cereus*	m28	Honey - Argentina	N/A
*Bacillus cereus*	m31	Honey- Argentina	N/A
*Bacillus cereus*	mv33	Honey- Argentina	KU230015.1
*Bacillus cereus*	cm37	Honey- Argentina	N/A
*Bacillus cereus*	mv39b	Honey- Argentina	N/A
*Bacillus cereus*	mv41x	Honey- Argentina	N/A
*Bacillus cereus*	mv54	Honey- Argentina	N/A
*Bacillus cereus*	m54	Honey- Argentina	N/A
*Bacillus cereus*	mv67	Honey- Argentina	N/A
*Bacillus cereus*	m73	Honey- Argentina	N/A
*Bacillus cereus*	mv73	Honey- Argentina	N/A
*Bacillus cereus*	mv75	Honey- Argentina	N/A
*Bacillus cereus*	mv76	Honey- Argentina	N/A
*Bacillus cereus*	mv77	Honey- Argentina	N/A
*Bacillus cereus*	mv78	Honey- Argentina	N/A
*Bacillus cereus*	mv79	Honey- Argentina	N/A
*Bacillus cereus*	mv80	Honey- Argentina	N/A
*Bacillus cereus*	m84	Honey- Argentina	N/A
*Bacillus cereus*	m85	Honey- Argentina	N/A
*Bacillus cereus*	mv86	Honey- Argentina	N/A
*Bacillus cereus*	mv87	Honey- Argentina	N/A
*Bacillus cereus*	m90	Honey- Argentina	N/A
*Bacillus cereus*	m91	Honey- Argentina	N/A
*Bacillus cereus*	m97	Honey- Argentina	N/A
*Bacillus cereus*	m105	Honey- Argentina	N/A
*Bacillus cereus*	mv114	Honey- Argentina	N/A
*Bacillus cereus*	mv117	Honey- Argentina	N/A
*Bacillus cereus*	cm117	Honey- Argentina	N/A
*Bacillus cereus*	cm118	Honey- Argentina	N/A
*Bacillus cereus*	m134	Honey- Argentina	N/A
*Bacillus cereus*	m139b	Honey- Argentina	N/A
*Bacillus cereus*	m143b	Honey- Argentina	N/A
*Bacillus cereus*	m143c	Honey- Argentina	N/A
*Bacillus cereus*	m157	Honey- Italy	N/A
*Bacillus cereus*	m158	Honey- Argentina	N/A
*Bacillus cereus*	m163a	Honey- Argentina	N/A
*Bacillus cereus*	m167	Honey- Argentina	N/A
*Bacillus cereus*	m189	Honey- Argentina	N/A
*Bacillus cereus*	m193	Honey- Argentina	N/A
*Bacillus cereus*	m225a	Honey- Argentina	N/A
*Bacillus cereus*	m228	Honey- Argentina	N/A
*Bacillus cereus*	m243	Honey- Argentina	N/A
*Bacillus cereus*	m244	Honey- Argentina	N/A
*Bacillus cereus*	m248	Honey- Argentina	N/A
*Bacillus cereus*	m262	Honey- Argentina	N/A
*Bacillus cereus*	m267	Honey- Argentina	N/A
*Bacillus cereus*	cm281	Honey- Argentina	N/A
*Bacillus cereus*	m282a	Honey- Argentina	N/A
*Bacillus cereus*	m287a	Honey- Argentina	N/A
*Bacillus cereus*	m292	Honey- Argentina	N/A
*Bacillus cereus*	m296	Honey- Argentina	N/A
*Bacillus cereus*	m298	Honey- Argentina	N/A
*Bacillus cereus*	m305	Honey- Argentina	N/A
*Bacillus cereus*	m308	Honey- Argentina	N/A
*Bacillus cereus*	m309	Honey- Argentina	N/A
*Bacillus cereus*	m316	Honey- Argentina	N/A
*Bacillus cereus*	m365	Honey- Argentina	N/A
*Bacillus cereus*	m370	Honey- Argentina	N/A
*Bacillus cereus*	m383	Honey- Argentina	N/A
*Bacillus cereus*	m385	Honey- Argentina	N/A
*Bacillus cereus*	m387	Honey- Argentina	KP005455.1
*Bacillus cereus*	m388	Honey- Argentina	N/A
*Bacillus cereus*	m434	Honey- Argentina	KU230027.1
*Bacillus cereus*	m436	Honey- Argentina	N/A
*Bacillus cereus*	m437b	Honey- Argentina	N/A
*Bacillus cereus*	m438	Honey- Argentina	N/A
*Bacillus cereus*	m439	Honey- Argentina	N/A
*Bacillus cereus*	m444	Honey- Argentina	N/A
*Bacillus cereus*	m445b	Honey - Argentina	N/A
*Bacillus cereus*	LPcer1	Honeybee larvae- Argentina	KX431225.1
*Bacillus cereus*	MexB	Honey- Mexico	KU230012.1
*Bacillus cereus*	MexC	Honey- Mexico	KU230013.1
*Bacillus circulans*	ATCC 4515	ATCC	N/A
*Bacillus clausii*	Fr231	Honey- France	KU230014.1
*Bacillus clausii*	m448b	Honey- Brazil	KX685159.1
*Bacillus coagulans*	NRRL NRS 609	NRRL	N/A
*Bacillus firmus*	ATCC 8247	ATCC	N/A
*Bacillus licheniformis*	mv55	Honey-Argentina	KU230018.1
*Bacillus licheniformis*	mv68	Honey-Argentina	MF187633.1
*Bacillus licheniformis*	mv72	Honey- Argentina	N/A
*Bacillus licheniformis*	m112	Honey- Argentina	N/A
*Bacillus licheniformis*	NRRL B-1001	NRRL	N/A
*Bacillus megaterium*	m280	Honey- Argentina	N/A
*Bacillus megaterium*	m327	Honey- Argentina	MF187637.1
*Bacillus megaterium*	m344	Honey- Argentina	N/A
*Bacillus megaterium*	m373	Honey- Argentina	N/A
*Bacillus megaterium*	m435	Honey- Mexico	KU230028.1
*Bacillus megaterium*	m441	Honey- Argentina	N/A
*Bacillus megaterium*	m458	Honey- Brazil	N/A
*Bacillus megaterium*	NRRL B-939	NRRL	N/A
*Bacillus mycoides*	m336	Honey- Argentina	MF187638.1
*Bacillus mycoides*	m425	Honey- Argentina	N/A
*Bacillus mycoides*	ATCC 10206	ATCC	N/A
*Bacillus pumilus*	mv41aA	Honey- Argentina	MG366818.1
*Bacillus pumilus*	mv49b	Honey- Argentina	KU230016.1
*Bacillus pumilus*	mv74	Honey- Argentina	MF972935.1
*Bacillus pumilus*	mv81	Honey- Argentina	KU230019.1
*Bacillus pumilus*	m108	Honey- Argentina	N/A
*Bacillus pumilus*	m116	Honey- Argentina	KU230020.1
*Bacillus pumilus*	m157	Honey- Italy	N/A
*Bacillus pumilus*	m187	Honey- Argentina	N/A
*Bacillus pumilus*	m225b	Honey- Argentina	N/A
*Bacillus pumilus*	m288	Honey- Argentina	MF187635.1
*Bacillus pumilus*	m330	Honey- Argentina	MF187646.1
*Bacillus pumilus*	m335	Honey- Argentina	N/A
*Bacillus pumilus*	m339	Honey- Argentina	MG366884.1
*Bacillus pumilus*	m350	Honey- Argentina	KU230023.1
*Bacillus pumilus*	m354	Honey- Argentina	N/A
*Bacillus pumilus*	m357	Honey- Argentina	MF187634.1
*Bacillus pumilus*	m358	Honey- Argentina	MG345110.1
*Bacillus pumilus*	m360	Honey- Argentina	MF187636.1
*Bacillus pumilus*	m363	Honey- Argentina	KU230024.1
*Bacillus pumilus*	m414	Honey- Argentina	KU230026.1
*Bacillus pumilus*	ATCC 7061^T^	ATCC	AY876289.1
*Bacillus subtilis*	m11	Honey- Argentina	N/A
*Bacillus subtilis*	m13	Honey - Argentina	MF187645.1
*Bacillus subtilis*	m45	Honey- Argentina	N/A
*Bacillus subtilis*	cm45	Honey- Argentina	MF187639.1
*Bacillus subtilis*	mv49a	Honey- Argentina	N/A
*Bacillus subtilis*	mv51	Honey- Argentina	N/A
*Bacillus subtilis*	mv53b	Honey- Argentina	N/A
*Bacillus subtilis*	mv63	Honey- Argentina	N/A
*Bacillus subtilis*	mv64	Honey- Argentina	N/A
*Bacillus subtilis*	mv65	Honey- Argentina	N/A
*Bacillus subtilis*	mv66	Honey- Argentina	N/A
*Bacillus subtilis*	mv70	Honey- Argentina	N/A
*Bacillus subtilis*	mv71	Honey- Argentina	N/A
*Bacillus subtilis*	m107	Honey- Argentina	N/A
*Bacillus subtilis*	m117	Honey- Argentina	N/A
*Bacillus subtilis*	m119	Honey- Argentina	N/A
*Bacillus subtilis*	m191	Honey- Argentina	MF187644.1
*Bacillus subtilis*	m192	Honey- Argentina	N/A
*Bacillus subtilis*	m197	Honey- Argentina	N/A
*Bacillus subtilis*	m291b	Honey- Argentina	N/A
*Bacillus subtilis*	m329	Honey- Argentina	KU230021.1
*Bacillus subtilis*	m334	Honey- Argentina	KU230022.1
*Bacillus subtilis*	m347	Honey- Argentina	KP177515.1
*Bacillus subtilis*	m351	Honey- Argentina	KP177516.1
*Bacillus subtilis*	m384	Honey- Argentina	N/A
*Bacillus subtilis*	m386	Honey- Argentina	N/A
*Bacillus subtilis*	m389	Honey- Argentina	N/A
*Bacillus subtilis*	m392	Honey- Argentina	MF187640.1
*Bacillus subtilis*	m412	Honey- Argentina	N/A
*Bacillus subtilis*	NRRL B-543	NRRL	N/A
*Bacillus thuringiensis*	ATCC 10792^T^	ATCC	D16281.1
*Bacillus thuringiensis*	m5	Honey - Argentina	N/A
*Bacillus thuringiensis*	mv50b	Honey - Argentina	KU230017.1
*Bacillus thuringiensis*	m391	Honey - Argentina	N/A
*Bacillus thuringiensis*	m395	Honey- Argentina	KU230025.1
*Bacillus thuringiensis*	m401	Honey- Argentina	N/A
*Brevibacillus borstelensis*	m348	Honey- Argentina	MF187641.1
*Brevibacillus borstelensis*	RC	Honey- Argentina	KP177514.1
*Brevibacillus brevis*	ATCC 8246	ATCC	N/A
*Brevibacillus laterosporus*	CCT 0031	CCT	N/A
*Brevibacillus laterosporus*	BLAT169	Honeybee larvae - Argentina	KX102627.1
*Brevibacillus laterosporus*	BLAT170	Honeybee larvae - Argentina	KX431223.1
*Brevibacillus laterosporus*	BLAT171	Honeybee larvae - Argentina	KX431224.1
*Lysinibacillus fusiformis*	mv119	Honey- Argentina	MG004185.1
*Lysinibacillus sphaericus*	ATCC 245	ATCC	N/A
*Lysinibacillus sphaericus*	m533	Honey- Argentina	MG001492.1
*Lysinibacillus sphaericus*	LMDZA	Honeybee larvae - Argentina	MG004191.1
*Paenibacillus alvei*	NRRL B-383	NRRL	N/A
*Paenibacillus alvei*	mv82	Honey- Argentina	MF187643.1
*Paenibacillus alvei*	m291a	Honey- Argentina	MF187632.1
*Paenibacillus alvei*	m420	Honey- Argentina	MF187642.1
*Paenibacillus amylolyticus*	NRRL B-14940	NRRL	N/A
*Paenibacillus apiarius*	ATCC 29575	ATCC	N/A
*Paenibacillus larvae* ERIC I	ATCC 9545^T^	ATCC	NR_118956.1
*Paenibacillus larvae* ERIC IV	ATCC 13537^T^	ATCC	KT363749.1
*Paenibacillus larvae* ERIC I	PL38	Honeybee larvae-Argentina	N/A
*Paenibacillus larvae* ERIC I	PL45	Honeybee larvae- France	N/A
*Paenibacillus larvae* ERIC I	PL58	Honeybee larvae- Sweden	N/A
*Paenibacillus larvae* ERIC II	SAG 290	Honey - Unknown	N/A
*Paenibacillus larvae* ERIC II	SAG 10367	Honey- Unknown	CP020557
*Paenibacillus larvae* ERIC II	SAG 10754	Honey- Unknown	N/A
*Paenibacillus polymyxa*	NRRL B-510	NRRL	N/A
*Rummeliibacillus stabekisii*	mv111	Honey- Argentina	MF972934.1

ATCC: American Type Culture Collection, USA; CCT: Colleçao de Culturas Tropical, Brazil; NRRL: Northern Utilization Research and Development Division, USA; SAG: Servicio Agrícola Ganadero, Chile.

N/A: Not applicable.

**Table 2:** Accession numbers of 16 S rRNA sequences from Type cultures used for sequence analysis.

Species	Strain	Accession number
*Bacillus amyloliquefaciens*	NBRC 15535	NR_112685.1
*Bacillus badius*	ATCC 14574	X77790.1
*Bacillus cereus*	ATCC 11778	NR_074540.1
*Bacillus circulans*	ATCC 4513	AY724690.1
*Bacillus clausii*	DSM 8716	X76440.1
*Bacillus coagulans*	ATCC 7050	DQ297928.1
*Bacillus firmus*	NBRC 15306	NR_112635.1
*Bacillus flexus*	IFO15715	NR_024691.1
*Bacillus licheniformis*	ATCC 14580	NR_074923.1
*Bacillus megaterium*	IAM 13418	D16273.1
*Bacillus mycoides*	ATCC 6462	NR_115993.1
*Bacillus niabensis*	4T19	AY998119.2
*Bacillus pumilus*	ATCC 7061	AY876289.1
*Bacillus simplex*	DSM 1321	AJ439078
*Bacillus subtilis*	DSM 10	JQ424889.1
*Bacillus thuringiensis*	IAM 12077	D16281.1
*Bacillus xiamenensis*	MCCC 1A00008	NR_148244.1
*Brevibacillus borstelensis*	DSM 6347	AB112721
*Brevibacillus brevis*	NBRC 15304	NR_041524.1
*Brevibacillus centrosporus*	NRRL NRS-664	NR_043414.1
*Brevibacillus formosus*	DSM 9885	AB112712.1
*Brevibacillus laterosporus*	IAM 12465	D16271
*Lysinibacillus fusiformis*	DSM 2898	AJ310083.1
*Lysinibacillus sphaericus*	ATCC 14577	NR_115724.1
*Paenibacillus alvei*	DSM 29	AJ320491
*Paenibacillus amylolyticus*	NRRL NRS-290	D85396.2
*Paenibacillus apiarius*	NRRL NRS-1438	NR_118834.1
*Paenibacillus larvae* subsp*. larvae*	ATCC 9545	NR_118956.1
*Paenibacillus larvae* subsp. *pulvifaciens*	ATCC 13537	KT363749.1
*Paenibacillus macerans*	IAM 12467	NR_040886
*Paenibacillus polymyxa*	DSM 36	AJ320493.1
*Rummeliibacillus stabekisii*	NBRC 104870	NR_114270
*Micrococcus luteus* (outlier)	DSM 20030	AJ536198.1

**Table 3:** Colony appearance and growth of selected strains tested in HiCrome Bacillus agar.

Species		Strain/Isolate designation	Colonies in HiCrome Bacillus Agar	Ecometric Code	
				HiCrome Control	Bacillus medium
*Bacillus amyloliquefaciens*		m39		5	5
*Bacillus badius*		ATTC 14574		3.8	3.4
*Bacillus cereus*		ATCC 11778		5	5
*Bacillus cereus*		m388		5	5
*Bacillus circulans*		ATCC 4515		4	5
*Bacillus clausii*		FR231		1.6	5
*Bacillus coagulans*		NRRL NRS 609		5	5
*Bacillus firmus*		ATCC 8247		5	5
*Bacillus licheniformis*		NRRL B-1001		5	5
*Bacillus megaterium*		NRRL B-939		4.6	5
*Bacillus mycoides*		ATCC 10206		4	5
*Bacillus pumilus*		ATCC 7061		5	5
*Bacillus subtilis*		m191		5	5
		NRRL B-543		4	5
*Bacillus thuringiensis*		ATCC 10792		5	5
*Brevibacillus borstelensis*		RC		4.2	5
*Brevibacillus brevis*		ATCC 8246		4.4	5
*Brevibacillus laterosporus*		CCT 0031		2.2	5
*Lysinibacillus fusiformis*		mv119		3.8	5
*Lysinibacillus sphaericus*		ATCC 245		1.4	5
*Paenibacillus alvei*		NRRL B-383		1	5
*Paenibacillus amylolyticus*		NRRL B-14940		4	5
*Paenibacillus apiarius*		ATCC 29575		4	5
*Paenibacillus larvae*	ERIC I	ATCC 9545		2	5
	ERIC IV	ATCC 13537		2	5
	ERIC II	SAG 290		3.8	5
*Paenibacillus polymyxa*		NRRL B-510		5	5
*Rummeliibacillus stabekisii*		mv111		5	5

## 2 Experimental design, materials, and methods

A collection of 197 bacterial isolates of *Bacillus, Brevibacillus, Lysinibacillus, Paenibacillus,* and *Rummeliibacillus* belonging to different species that have been reported in honey [1], [2], [3], [5], [7], [10], [12], [13], [14], [15] were screened for their abilities to grow and colony appearance and color, and any substrate color change by using HiCrome Bacillus agar. The collection includes 167 isolates from honey samples from different geographical areas including Argentina, Brazil, France, Italy, and Mexico; 9 isolates from honeybee larvae from different geographical areas including Argentina, France and Sweden; and 21 strains from Culture Collections used for comparison and quality control. Bacteria were maintained as stock cultures at −80 °C in the correspondent broth medium, Müller-Hinton Broth, yeast extract, potassium phosphate, glucose, and pyruvate (MYPGP) [4] or Brain heart infusion (BHI) plus 20% glycerol (v/v). For short-term storage, the strains were kept at 4 °C in screw-capped vials containing MYPGP or BHI semi-solid (0.4% agar).

Bacterial smears stained by Schaeffer-Fulton technique were examined for the presence and location of spores within cells, as well as for the size and shape of vegetative cells [9], [11]. Also, the presence of unstained globules in the cytoplasm [6], [9], [11] was examined by phase contrast microscopy (Leica, ICC50).

Bacterial cultures were also tested by catalase reaction, anaerobic growth, nitrate reduction, Voges-Proskauer reaction (VP), pH in VP broth, indol and urease production, mannitol, L-arabinose, and citrate utilization, starch and gelatin hydrolysis, decomposition of tyrosine, growth in 7% and 10% of NaCl and at different temperatures (30-37-50 and 55 °C) according to standard protocols [6], [9], [11].

The Ecometric technique was used for comparative evaluation of HiCrome Bacillus agar and the correspondent control media (BHI or MYPGP). Overnight cultures were adjusted to 0.5 Mc Farland in sterile distilled water. One loop of 10 μl of each suspension was sequentially diluted from streak to streak onto each medium by inoculating 21 streaks (5 per quadrant and 1 in the center). Growth on the plates was recorded as a score. Readings were presented as absolute growth indices with possible values of 0–5, where 0 is an absence of growth in any streak and 5 was the maximum score obtained when all of the streaks in the four quadrants and also the last streak showed visible bacterial growth [2], [8]. Twenty-eight bacterial strains with different colony types (Table 2) were used for the evaluation. Plates were inoculated and incubated in duplicate for 24–48 h at 37 °C. Scores for HiCrome and control plates were compared to estimate the degree of inhibition due to the chromogenic mixture (Table 3, Fig. 2, Fig. 3).

The identity of selected strains (n = 56) was confirmed by sequencing the 16S rDNA. Universal eubacterial primers used for 16S rDNA sequence analysis were 27f (5′-AGAGTTTGATCMTGGCTCAG - 3′) and 1492r (5′-TACGGYTACCTTGTTACGACTT- 3′).

For purification of PCR products the following enzymatic procedure was used: The mixture contained 0.5 μl Antarctic phosphatase buffer (NEB, Migliore Lacaustra, Argentina), 0.6 μl Antarctic phosphatase (NEB, Migliore Lacaustra, Argentina), 0.6 μl Exonuclease I (NEB, Migliore Lacaustra, Argentina), 4 μl unpurified PCR product and 3.3 μl double distilled sterile deionized water. The Thermal cycler protocol consisted of one step of 37 °C for 20 min and the second of 80 °C for 20 min.

The quality and quantity of PCR products were assessed by gel electrophoresis (1 μl/1.6% agarose/molecular weight marker QuantiMarker, Promega, Argentina) and DNA concentration was estimated by using a Genova Nano spectrophotometer (JenWay). The purified PCR products of approximately 1,400 bp were sequenced by the dideoxy termination method by the commercial services of Macrogen Inc. (Seoul, Korea) or Unidad de Genómica, Instituto de Biotecnología, CICVYA - INTA (Hurlingham, Argentina). Sequence assembly and contig editing were performed by using CodonCode Aligner software (Codon Code Corporation, MA, USA). The partial sequences obtained were subjected to both Blast-N (http://www.ncbi.nlm.nih.gov), and EZBiocloud (http://www.ezbiocloud.n) search to identify sequences with the highest similarity by comparison only with sequences obtained from Type Cultures [2].
